# Tribute to Kenneth J. Breslauer

**DOI:** 10.3390/life12091325

**Published:** 2022-08-27

**Authors:** Tigran V. Chalikian

**Affiliations:** Department of Pharmaceutical Sciences, University of Toronto, Toronto, ON M5S 3M2, Canada; t.chalikian@utoronto.ca

It is an exciting experience to serve as guest editor for a Special Issue celebrating the 75th birthday of Professor Kenneth J. Breslauer. For more than five decades, Ken Breslauer has been one of the giants shaping the field of biopolymer thermodynamics. After doctoral studies in the laboratory of Prof. Julian Sturtevant and postdoctoral training in the laboratory of Prof. Ignacio Tinoco, Ken joined the Department of Chemistry of Rutgers University. That was in 1974, and he has remained loyal both to Chemistry and to Rutgers ever since.

Ken’s lasting impact on molecular biophysics has been especially prominent in his fundamental quest to elucidate the energetic determinants of DNA polymorphism, drug–DNA interactions, DNA repair, and, more recently, the molecular mechanisms of genetic diseases associated with uncontrolled triplet expansion. In retrospect, Ken’s pivotal and pioneering studies, particularly into the nearest-neighbor-based thermodynamics of DNA stability and the differential energetics of drug binding to compositionally similar but structurally and hydrationally distinct DNA duplexes, emerge as high points in the progress in our understanding of the physical principles governing the structure and function of DNA.

Ground-breaking work in the Breslauer lab convincingly showed that the association of the quintessential minor groove binder netropsin with the poly(dA)poly(dT) and poly(dAdT)poly(dAdT) duplexes, while being characterized by nearly identical binding free energies, has dramatically different enthalpic and entropic origins [1]. Ken’s seminal paper on the nearest-neighbor thermodynamics of DNA, published in *PNAS* in 1986, inspired wide-spread endeavors that continue to this day [2]. Those efforts have resulted in the development of nearest-neighbor databases for computing the full thermodynamic profiles of the duplex-to-single-strand transitions of any duplex DNA, including canonical B-DNA, mismatched DNA, mutagenic DNA lesions, and hairpins, under different environmental conditions (for summary, see ref. [3]). More recently, his studies have provided novel thermodynamic insights into the evolution of the genetic code [4] and resulted in the discovery of “rollamers” [5]. The latter represent dynamic polymorphic DNA structures that may explain the inception and progression of triplet expansion-based genetic diseases and suggest possible avenues for their treatment.

For decades, the Breslauer lab has been a conceptual powerhouse in the development of thermodynamic insights into the functioning of DNA. As such, it has been a hub for collaboration among, and a destination for visits from, highly accomplished scientists from around the world. I was fortunate to spend five years between 1992 and 1997 in this vibrant atmosphere. Ken’s mentorship was unobtrusive and unforced, giving you all the freedom you needed to test your ideas. At the same time, he was generously supportive should you need his guidance to overcome a seemingly insurmountable obstacle. More than anything, he was a constant and wonderful example of a superb scholar and effective principal investigator. I learned a great deal from him. I learned the importance of looking broadly into the scientific problem at hand and of thinking outside the limits of a specific experimental technique, employing to that end a combination of techniques, each offering a unique insight into the problem.

I gratefully acknowledge that, throughout my subsequent career as an independent researcher, I felt Ken’s gentle support and guiding attention. Recently, helping my son with his biology class, I recounted the Hershey–Chase experiment in which DNA, and not the protein, was shown to carry the infectiousness of the phage. It occurred to me that I knew first-hand that DNA is infectious. Before joining Ken’s lab, proteins had been the center of my research interests. Ken infected me with his enthusiasm and passion for DNA. Twenty-five years later, I still retain that infection.

This Special Issue is a tribute to Ken’s influential presence in the field of DNA biophysics. It comprises 15 papers that broadly encompass the current state-of-the-art in the field. They include experimental and theoretical studies into the stability and interactions of canonical and noncanonical DNA structures. In some respects, these studies parallel, complement, and extend concepts Ken Breslauer articulated decades ago and has been developing ever since.

Below, we present, in alphabetical order, the testimonials and personal accounts from people who have known Ken at different stages of his career as a mentor, a colleague, or a friend.

## 1. Robert Boikess, Professor

When I was Ken’s department chair, and even afterwards, looking after his best interests was always one of my major concerns. When he was up for promotion and tenure, some senior faculty were concerned that he didn’t have enough funding. I assured them that a lot more would be coming (something of an understatement). Over the years, his rare combination of skills as a research scientist and as a scientific administrator attracted the attention of other universities. Fortunately, I was able to convince the Rutgers administration to do what was necessary to keep him at Rutgers. These actions were something that neither I, nor anyone else at Rutgers, ever regretted.

## 2. Walter A. Dickerhof, Lab Technician

My employment under Ken at Wright-Riemann Laboratories was the first full-time position in my field of study after having graduated college. Despite his impressive stature at the University, Ken proved to be remarkably approachable, as well as extremely welcoming to those new to his group thereby facilitating a seamless integration into same. Though the essence of our affiliation was implicitly professional, Ken possessed a genuine sincerity underpinning this professionalism and serving to foster a relationship at a more personal level while at the same time preserving his authority.

Years after I had left the University, I contacted Ken regarding a reference for potential employment and was pleasantly surprised as to how enthusiastically receptive he was after so many years; this moment being void of any awkwardness and having a feel as if it were days, not years, which had passed since our last conversation. That communication, as well as those following during the brief period thereafter, had this old-friend kind of vibe to them. For this, I cannot find words sufficient but can only state, simply, that it speaks to his character, to wit: typically, it is the employer who expects loyalty from their subordinates; nevertheless, nearly two decades later, still I had Ken’s support. This was an exceptional act of loyalty, one by which I felt flattered and, even more so, humbled.

Inarguably intrinsic to Ken’s nature, the afore-described attributes instilled in me a visceral sense of belonging as well as a strong feeling of pride to be a member of his research group; sentiments which have persisted with me until this very day.

Thank you, Ken, for your kindness, your generosity and all that you had done for me then and what you have done for me since.

Happy Birthday, my friend.

## 3. Ann Doeffinger, Administrative Assistant

Simply having the opportunity to wish “75th Happy Birthday” to—and help celebrate the life of—such a Distinguished Professor and a gentleman as Dr. Kenneth Breslauer is in itself a gift.

It has been an immense honor and pleasure to work in the Chemistry Department alongside one of its most pre-eminent professors, as he is truly a remarkable person in every respect.

“I have come to believe that a great professor is a great artist and that there are few as there are any other great artists. Teaching might even be the greatest of arts since the medium is the human mind and spirit”

—*John Steinbeck*

## 4. Dick Foley, Dean, Professor Emeritus

As Dean of Arts and Science at Rutgers for a decade, I worked with Ken and saw first-hand what a remarkable academic administrator he is. “Remarkable” understates the case. His record of successes at Rutgers is unprecedented. It was during that decade that he oversaw a reorganization of the life sciences and established the Division of Life Sciences, but it was anything but an ordinary reorganization. Every university I have been associated with has an exaggerated sense of itself as uniquely complex, but at Rutgers in those years, my love for the place notwithstanding, this was no exaggeration. Its unusual history and administrative structures had produced a confusing university-wide tangle of schools, departments, and institutes that duplicated each other’s research and teaching portfolios in the life sciences and did so, frankly, with notably different degrees of quality. The task of unsorting and then reassembling this jumble demanded equal measures of academic vision, political skill, and sheer tenacity. It was a herculean task which Ken pulled off in ways that no one else could have approximated, the eventual result being a well-integrated collection of prospering departments, centers, institutes, state-of-the-art buildings, core facilities, and the list goes on and on. Impressive as all this is, the most important of Ken’s successes over the years are the result of his eye for intellectual talent and ability to attract it. Time after time he competed successfully against far better endowed universities to recruit and retain top faculty. He did so with his characteristic persistence but also ingenious arrangements, all done with a fraction of the resources of the competition. With apologies to Churchill, never has so much been accomplished with so little. It was exciting to be Ken’s partner and co-conspirator during that decade, but it was also just great fun. Little in life is better than working on undeniably valuable projects with someone you enormously admire but also hugely enjoy.

## 5. Barbara Gaffney, Lecturer

I was a Lecturer in the Douglass College Department of Chemistry when, in 1974, our esteemed chair, Stan Mandeles, was fortunate in recruiting Ken Breslauer as a young Assistant Professor. Stan had himself been recruited to build a cutting-edge research department at Douglass that was to be focused at the interface of chemistry and life sciences (this was well before the 1981 reorganization that combined the various college chemistry departments into a single unit at Rutgers Wright Labs). Our small Douglass faculty were friendly and collegial, and Ken fit right in, with his great charm and wit. He was a natural teacher, so he was exceedingly popular with the outstanding Douglass undergraduates. Research funding was scarce, so Stan’s new hires were all encouraged to seek external funding, at which Ken showed exceptional skill. With great care, Ken set up his biophysical lab with good instrumentation: his first spectrophotometer and calorimeter. He mentored several graduate students but depended primarily on post-doctoral fellows. After the 1981 reorganization of the college chemistry departments, Ken would soon move his operation to Wright Labs, where it flourished. At Douglass, he had overseen the medical technology program, so perhaps it was natural that at Busch he would again choose to combine research with administration. In 1985, he became the Associate Dean of Life Sciences, and the rest, as they say, is history.

## 6. Craig A. Gelfand, CEO

It was a tremendous privilege to have been part of the Breslauer lab in the late 1990s, and only later could I look back with full appreciation upon the spectrum of scientific and career learnings. It is impossible to distill 4 years into a few paragraphs. To my non-scientist friends, I often tell silly one-liners like the fact that a lot of us developed an unhealthy addiction to caffeine to offset the slow pace at which a DSC generates data. I will leave deeply scientific topics to my esteemed colleagues, the amazing people that continued to work and thrive in the thermodynamics community. While my career path moved me away from thermodynamics as a main research topic, the lessons I learned in the Breslauer lab, much like the science of thermodynamics itself, always seem apply to the projects that I am dealing with. So, I would like to take this opportunity to thank Dr. Ken Breslauer for his patience in teaching an excited kid that had a lot to learn and to describe a few of the key universal lessons that I was privileged to have learned from a wonderful mentor.

One lesson that applied over-and-over during my career was that hypothesis-driven experiment planning needed to be balanced with flexibility to let resulting data tell their own story—in essence, do not let your preconceived notions interfere with thorough and thoughtful interpretation of your data. A few aspects of my own work as a post-doctoral fellow and research associate in Ken’s lab are perhaps a small example of this, but the body of work coming from the Breslauer lab at large serves as tribute to the breadth of inventiveness that this approach enables: the data themselves are the main storytellers; successful researchers listen to those stories, communicate them by publication or presentation, and then help write the next chapter of the story by devising new experiments based on the prior data. I write this knowing that to many this will seem superficial (e. g., “that’s how science is supposed to be done”), but throughout the years of my own career that followed, I observed others (and occasionally caught myself) making the mistake of preconceiving desired outcomes, often leading to derailed projects; so, it’s a true career benefit that I received this learning during my post-doc years. Productive scientists succeed by being clever, flexible, and patient observers and good stewards of data. Among the achievements that I take most pride in from my own work are the few times when I was asked to solve ‘project-killing messes’ merely by letting each problem tell itself to me first through the existing data, then navigating to successful solutions.

A correlated lesson is the ability to tell those stories. Ken is a gifted communicator, able to convert the complicated into the understandable, capable of presenting, seemingly effortlessly, to a wide spectrum of people from deeply technical to lay audiences and, even more uniquely, with flair and enthusiasm. There is an intrinsic career barrier that I think is a particularly acute burden in complex fields such as thermodynamics: a communication ‘activation energy barrier’ that needs to be overcome. The work of any scientist can be rendered essentially irrelevant if we cannot communicate it, sometimes with the paradox that the more unusual or novel the story that needs to be told, the higher the burden for precise and concise communication. Ken is a master, and it is clear that he took extra effort to mentor us lab folk to be better communicators, in parallel with teaching us the science. I clearly recall being exposed to the equally important yet somewhat different skills required to prepare effective oral presentations (through hours of editing and re-working of slides—especially in the prior era when physical transparencies and slides needed to be finished well ahead of time) and written works (I recall at least a few times that Ken somehow managed to write more by hand in red ink in the margins and on backs of pages than I had typed in preparing a draft). These are lessons that are more easily appreciated in retrospect, and skills that I am certain benefitted my career. My Ph. D. advisor, Dr. Joyce Jentoft, had a similar passion for teaching the critical skill of communication of science, so I was doubly fortunate of having two mentors drive this topic. I hope that I have honored the importance of these lessons by passing them on to scientists that I trained and worked with, helping support their careers in the way that my mentors helped me.

I look forward to the privilege of continuing to learn from Ken and from my continued association with his lab.

## 7. Vera Gindikin, Research Associate

I am grateful for this opportunity to express the appreciation I have had for many years now for Dr. Breslauer and to convey how lucky I feel to have been working in his lab.

Dr. Breslauer, first and foremost, is an incredible, brilliant, and highly devoted scientist, who never ceased to amaze me with his extraordinary mind, vast knowledge, and unique perspective on things. These qualities are well known and appreciated by many who have met and collaborated with Prof. Breslauer, quite a few of whom are contributing to this publication. However, I am also fortunate to have had a personal perspective of Dr. Breslauer by having been a part of the Breslauer lab for the past three decades.

I met Dr. Breslauer for the first time a few years before I joined Rutgers University, when I visited the US while still being an undergraduate student majoring in Biochemistry and Molecular Biology. I was planning to apply to graduate schools and wanted to talk to faculty at Rutgers chemistry department. Dr. Breslauer not only agreed to meet with me but spent a long time of what I am sure was a very busy day talking to me and then introducing me to some of the other faculty members. I also remember how incredible comfortable he made me feel; it was all very surprising and unusual. I had never met people like him before, and now, more than 30 years later, I can honestly say that I have never met people like him since.

I consider myself very fortunate for having joined Ken’s lab as a graduate student in 1992 and to be able to continue working in the lab after graduation. Not only did I have the luxury of scientific research flexibility and the input and care of a wonderful scientific advisor, but I also always had incredible personal support from Dr. Breslauer during some difficult times in my life. Dr. Breslauer always found kind words and great solutions to help me, and it made me feel stronger because I felt that support. I think it is an incredible gift to feel such support from one’s advisor, which only a very few lucky people get to experience. I am among these few lucky ones, and I am eternally grateful.

Happy Birthday from one very grateful and appreciative “last graduate student”!

## 8. Arthur P. Grollman, MD, Professor

The Shakespearian theme “What’s past is prologue”, coupled with the quest of “How small molecules do great things” that guides research in the Zickler Laboratory of Chemical Biology (LCB), epitomizes my 30-year collaboration with Ken Breslauer and Francis Johnson, Director of the Division of Medicinal Chemistry at Stony Brook. Our collaboration continues apace on Ken’s 75th birthday as it did when the three of us were considered young Turks.

In 1993, funding was obtained from the National Cancer Institute to support a Program Project grant (PPG), “Exocyclic DNA Adducts and Oxidative DNA Damage.” The central theme of this multidisciplinary program involved relationships between molecular structure, energetics, and biological activity. The integration of these factors proved to be central to understanding the molecular mechanism(s) of DNA replication, DNA repair, and mutagenesis. At that time, the mapping of energy landscapes that link structures with biological functions were entirely lacking. Breslauer’s research and databases proved critical in bridging this experimental and conceptual gap. His group’s seminal research was a unique feature of our PPG, contributing to enthusiastic reviews and continuous NIH funding for this research program for more than 20 years.

The site-specific introduction of single lesions into DNA, using innovative synthetic methods developed in the Johnson lab at Stony Brook, provided modified DNAs with which Breslauer and his talented research associates at Rutgers generated novel energetic insights and concepts, designed to complement structure-based interpretation of biological processes [3].

As a physician-scientist, I was acutely aware of the paucity of drugs considered safe and effective in treating human viral disease. Accordingly, when I initiated a program on rational antiviral drug design, supported by private philanthropy, it was clear that Breslauer and his group were among the few who could meet the exacting goals of such a program. Ken serves on the three-person Executive Committee that guides our blue-ribbon consortium. Our first report (on the active principle of ATA) appears in this Festschrift. As orally effective drugs are urgently needed to combat SARS-CoV-2, we have chosen to repurpose emetine as an antiviral, using low doses to minimize the well-known side effects of this established amebicide. Ken’s extensive experience with drug development, together with administrative skills honed over decades, which are summarized in this Editorial, are playing an important role in overcoming the bureaucratic roadblocks associated with our drug development research.

As a personal friend and colleague, Ken has an upbeat attitude toward others diplomatically helping where he can. This is supported by an imaginative capability to solve problems through useful suggestions, always with a light touch and a wonderful sense of humor. He is a scientist for all reasons in all seasons.

## 9. Martha Haviland, Professor

I first met Ken Breslauer when he was the Dean, Division of Life Sciences, Linus C. Pauling Distinguished Professor, and Vice President, Health Science Partnerships, and I was the Director of Undergraduate Advising for the Division of Life Sciences. As I understand the history, it is Ken’s vision and his ability to work with and motivate others that led to the development of the Division and, ultimately, the undergraduate program in life sciences at Rutgers. But it was not until I moved into the position of Director of the Undergraduate Instruction for the Division and reported directly to Ken that I truly understood his dedication to undergraduate education. As just one example, under his leadership and guidance, we revamped our introductory General Biology curriculum, received funding from NSF and the State of New Jersey for our efforts, and leveraged this external funding to secure internal funds to renovate undergraduate classroom laboratory spaces as well as increase the number of teaching assistant positions assigned to the Division. You might expect that a person with his responsibilities might not prioritize individual undergraduate students, but Ken ensured that every e-mail from a student was answered. Some of my fondest memories are when Ken attended undergraduate celebrations, whether they be research symposia, graduation events, or Rutgers Day. His smile is engaging, he always has his hand out to shake yours, and he made each student he spoke with, and their parents, know that they were heard and respected. Finally, on a personal note, Ken encouraged me and supported my growth as an academic and an administrator. He provided me with the autonomy and authority to make decisions and move forward, but I always knew he had my back and would step in to provide support if asked.

## 10. Roger Jones, Professor Emeritus

Ken has always been a visionary.

I first met Ken in 1977, upon my hire into the Chemistry Department of Douglass College, which is part of Rutgers University. Ken was a few years ahead of me in the Assistant Professor ranks, but he was already influential in the department. Ken was universally liked by both faculty and students. He never missed an opportunity to assist, support, and encourage his students to do their best in addition to being a loyal and professional colleague. We had both been hired as part of the plan of the Chair, Stan Mandeles, to add nucleic acids research to the department, and we quickly developed a lifelong friendship. Nucleic acids research was not common in chemistry departments at the time and was not represented in the much larger Rutgers College department. It happened that the two departments were merged in 1981, and it was my fate to come up for tenure in 1982. Likely, it was Ken’s eloquence that convinced the newly combined faculty that my synthetic nucleic acid program was worth promoting. He was always ready to help a colleague.

Ken’s long-standing goal was to grow life sciences at Rutgers, which he did with great success! From his time at Douglass College, and continuing through his university administrative positions, Ken proved as adept at raising Rutgers funds to support his life science goals as he was at raising NIH funds to support his research program. In my 42 years at Rutgers, Ken is unique in his ability to sustain a high-quality research program while simultaneously a highly successful series of substantial administrative positions.

“The qualities of a great man are vision, integrity, courage, understanding, the power of articulation, and profundity of character”

—*Dwight Eisenhower*

## 11. Mike Kiledjian, Professor

Happiest of birthdays Ken!

Thank you for all your tireless dedication and support of the Life Sciences at Rutgers University. Your vision to coalesce life science research within the School of Arts and Sciences into a single division and build a first-rate research program was visionary, and we are all better now because of your forward thinking.

Ken and I first met when I was an undergraduate student at Rutgers working in a lab within the Department of Chemistry. He and my research advisor were colleagues, and Ken was a valuable source of knowledge and guidance. A decade later in 1995, Ken was instrumental in recruiting me back to Rutgers as a junior faculty member in what is now the Department of Cell Biology and Neuroscience (CBN). I have had the pleasure of working closely with Ken in his role as Dean of the Division of Life Science. Ken’s tireless efforts to establish, foster, and lead the Division of Life Sciences at Rutgers is a testament to his commitment to scientific excellence.

On a personal level, I am grateful to Ken for his support and mentorship during my appointment as Chair of CBN in 2012. The CBN faculty and I are forever indebted to Ken for his unwavering support throughout the years that enabled us to recruit outstanding junior faculty and establish a superb foundation for the growth of the department. This momentum continues to this day and was initiated in large part to Ken’s support.

Cheers to 75 amazing years and to many, many more to come!

## 12. Elsa Klump, Artist, Widow of Professor Horst Klump

What do I recollect of the Ken Breslauer stories, with which my beloved Horst Klump entertained me? Of course, it was not academic. I am a sculptor, a listener, and an enthusiastic identifier with the lives, thinking, and products of others. This account is therefore second-hand creative hearsay.

How did Horst and Ken connect? To me they recognized each other as brilliant entrepreneurs and scientists, streetwise survivors who could compete, work, and play hard—both top academics with talents in many more directions, bordering on genius.

Horst was an Assistant Professor in the Dept of Physical Chemistry 2, and at times Acting-Head, at the University of Freiburg, Germany, when Ken Breslauer arrived in 1981/82 on a Humboldt Fellowship for post-doctoral research. There were complications though. Both the families of Ken’s father and mother, Breslauer and Schaeffer, had fled Germany during the Nazi regime, ahead of the Hitler horrors. Their home was on the East-Coast USA now, and Ken’s father had misgivings about his son’s return to Germany. So, the parents accompanied their very adult son—Ken Breslauer—back to Germany to assure his well-being, comfort, and safety.

Horst received the Breslauers and welcomed Ken into his family life with two young sons. For Horst it was an honor, and he formed a strong connection with Ken. They were both top researchers, breaking new ground; they understood and appreciated each other’s projects, even when one was more than a decade senior to the other. They were both keen sportsmen. Horst was a yachtsman, a tennis and soccer player, and skied in winter. Ken could have become a professional sportsman/baseball player. Together they had fun. As I see it, Ken is a team sports person, whereas Horst was into individual sports. Somehow, through Ken’s personality, Horst was roped in. The parents could leave their son behind in Horst’s trustworthy hands. In turn, Horst could trust Ken and eventually was even willing to entrust his lifetime research to him.

When Ken returned to academic life in the USA, they stayed in contact. Horst would visit the Breslauer family when on sabbatical or attending conferences in USA, first at Ken’s parental home in Queens and later near Rutgers, Ken now having two sons of his own. At first, Ken focused on research, but he quickly extended his talents into managing, developing, and expanding the status of life sciences at Rutgers, The State University of New Jersey, USA. He kept his research lab, entrusted to capable researchers, but most of his time was taken up running Rutgers.

In 2000, when Ken was a Linus Pauling Professor of Chemistry and the Dean and Director of Life Sciences at Rutgers, Horst (and I) came to Ken’s lab, initially for the first 3 months of Horst’s sabbatical year from UCT. Horst found this setup at Rutgers so conducive to his own research that (although he also planned periods at West Coast Universities) he preferred to stay for most of that year and, from 2002 on, returned annually for the allowed three-month stints for another 14 times. Horst, as a brilliant original researcher and talented lateral thinker, found it difficult when others would take his ideas to appropriate the work for themselves. However, Horst was willing to entrust Ken with his unpublished research accomplishments—“ploughing with other’s oxen”. Ken could comprehend the thrust of a thought direction, a research paper, facilitate possibilities and even repackage the presentation of a publication to make the work more accessible and marketable, but he would always acknowledge the creator/initiator/pioneer. The Breslauer family CAN write. So could Horst, but better in his mother tongue, German.

I got the impression, as an outsider in science, that Ken appointed scientists he trusted and then allowed them a measure of freedom to develop science projects that suited their own talents and personalities best. Jens Voelker, a former PhD student of Horst’s from UCT, now part of Ken’s group, put it this way: “This (research) could not be done without Ken’s support and his willingness to let us play”.

Nancy Ludowicki, PA, was Ken’s backbone and was hands on with Ken’s many Rutgers University projects. Nancy could achieve directives on many levels. I think Nancy’s capabilities were wide. Nancy equipped and installed us, for example, into our living quarters at Treetops Apartments at the edge of Rutgers’ “Ecological Forest”, where wildlife abounded. Apparently, the day before she died, when Ken visited Nancy in hospital, Nancy still gave Ken the where’s and whatall’s that she felt Ken needed to know about the tasks that she was leaving behind.

During Horst’s sabbaticals, Ken arranged for me to do sculpture at the Rutgers arts school lab on Livingston campus. What a privilege! I had interesting discussions on art with Ken Breslauer, and I was amazed at the depth of Ken’s understanding, insights, and advice upon explaining my sculptural art.

I worked in clay and wood and received all the support needed from Rutgers staff. How grateful I am. Thanks to this arrangement enabled by Ken I was able to exhibit with the art lecturers at the Gallery of Mason Gross School of Art and to connect with so many creatively different post-graduate students. I would like to believe I contributed to the development of their thought processes.

## 13. Ajar Kochar, MD, MHS

I had the privilege of first meeting Dr. Breslauer circa 2005–2006 as a young, highly impressionable Rutgers undergraduate student. At the time, stem cell research was very much in vogue as a potential treatment modality for a panoply of medical pathologies. However, the ethical dilemmas surrounding the use of embryonic stem cells was extremely politically charged. Rutgers, and New Jersey more broadly, were being considered as a central hub for a major stem cell research center. Rutgers was and remains a driving force for academic scholarship and community engagement. Rutgers is able to play this role in large part due to the inimitable leadership of icons like Dr. Breslauer. Dr. Breslauer, in partnership with another college mentor of mine—Dr. Wise Young—helped organize a series of community-based events focused on stem cell research.

I initially got to know Dr. Breslauer in helping to plan these stem-cell-research-based conferences. I vividly recall the first-time meeting with Dr. Breslauer in his office. I was simply awed by his inimitable presence as he radiated a unique combination of academic brilliance, administrative nous, and genuine compassion. Looking back, I had no business being anywhere near those conferences. I had at best a paltry understanding of the science let alone the complex interplay between the science and politics of high-level scientific investigation. Yet, Dr. Breslauer never let me or my classmates (including the incredibly talented Dr. Nakul Raykar, who is now a rock-star trauma surgeon/clinical researcher at Brigham and Women’s Hospital/Harvard Medical School) ever feel like our opinions were immaterial. In fact, he encouraged us drive the ship while skillfully shepherding us through the process. Under Dr. Breslauer’s mentorship, we were able to help execute a very well attended and insightful series of programs focused on stem cell research.

Much to my delight, after the completion of these stem cell research events, Dr. Breslauer continued to remain a key college mentor and role model. The transition from college to “what’s next” is always an incredibly stressful period. Dr. Breslauer played an integral role with my application to medical school, even going so far as to make phone calls to advocate on my behalf. I am confident that without his support I would not have ultimately landed at Brown Medical School, where I had a tremendous experience in medical training. Not only did Dr. Breslauer go above and beyond to support me and many others, but he was incredibly encouraging and nurturing during the process. Moreover, he provided a fantastic example on how to pursue a career in science employing steadfast dedication, passion, and a pinch of humor. Admittedly, I am still at the beginning phases of my own academic career—but I often think about Dr. Breslauer as a North Star role model to emulate.

I would be remiss not to mention that some of my most enjoyable experiences with Dr. Breslauer have been beyond the realm of academics. I was a senior when Rutgers beat Louisville in 2006. I recall celebrating with Dr. Breslauer a few days later and Dr. Breslauer explaining the downstream positive impacts of such a victory on the entire University community. More recently, I have relished celebrating the incredible accomplishments of our Men’s Basketball team successfully qualifying for the NCAA tournaments in back-back (really should be a third back…) years. Dr. Breslauer’s passion beyond science speaks to the Renaissance man that he is. I only hope I will continue to have the opportunity to learn from his incredible expertise and will conclude by simply saying: thank you for a lifetime of memories and mentorship.

## 14. Ernie Lepore, Board of Governors Professor

I have known Ken for decades now, and I have always thought of him as something like a big brother, probably because he always helps me get out of various messes I created; twice he bailed out two of my graduate students with summer funding when I forgot to secure the funding myself. One of them is at NYU now; the other is at Princeton. Thank you, Ken, from them both. Several times he’s allowed me to use the life sciences building for conferences I organized. This was especially helpful during the hurricanes we suffered. And the list goes on and on. Oddly, I even trust his judgment more than I trust the judgment of my own internist and his internist, too. His storehouse of knowledge is something to marvel—ranging from chemistry to life sciences to sports to academia to campus politics and, yes, even to medicine. To envy his long list of accomplishments would not only be presumptuous but would also deprive you of enjoying them. I have been pretty successful in moving Rutgers forward: building an internationally recognized department, hiring National Academy of Sciences members. But then there’s Ken—one building, two buildings, three buildings. Incredible. I sometimes wonder whether Rutgers has enough space to house all the buildings that Ken’s planning. But it would be remiss not to acknowledge the magic of his voice. Whenever I hear it, it brightens my day.

## 15. Charles Martin, Professor Emeritus

Ken’s extraordinary science will be clear from others who write here. Like many others, I followed his research and benefitted from his scientific achievements. I can also testify about as his skills as an administrator and program builder. In my opinion, much of expansion of the life sciences at Rutgers and the reunification of the medical school with Rutgers can be directly attributed to Ken’s efforts.

In 1996, Ken was appointed as the Dean of the new Division of Life Sciences within the School of Arts and Sciences. Ken asked me to assist him and got me appointed as the Director of the Bureau of Biological Research—it was at this point that I was able to closely watch and marvel at his administrative, social, and political abilities. Ken has a remarkable gift for bringing people together, extracting money and effort from them, and getting them to do big things. His first efforts were to raise money for recruiting and building. And what a job he did! Through his well-founded connections built during the first reorganization in the 1980s, he managed to acquire internal funds from various sources within the University. He also recognized that he would need to hire “rainmakers”—well-funded and nationally recognized scientists to build and head new departments and research centers. His first effort was to hire Wise Young to establish the Center for Collaborative Research in Neuroscience. Wise was a prominent NYU Neurobiologist who specialized in spinal cord injury research. Ken recruited Wise and supported his fund-raising efforts from sources ranging from the Keck Foundation (a USD 2.1 million grant), Wall Street bankers, pharmaceutical firms, you name it. We had many meetings, and I got to watch up close (with some “I didn’t know you could do that”, jaw-dropping amazement) as he and Wise organized and ran fund-raising events on Wall Street and at the Lincoln Center that were used to build the Center in a large space connected to Nelson Laboratories. This effort paid off very well in the early days of the Division and brought a lot of good press to the University and the Division of Life Sciences.

Ken’s second big effort was recruiting Jay Tischfield to establish the Department of Genetics and the Human Genetics Institute. Jay was a well-known human geneticist at the University of Indiana Medical School who had established a well-funded human cell repository. When Jay moved to Rutgers, he greatly expanded the repository and its funding base, which was then used to recruit top faculty and establish the impressive Department of Genetics that we see today. Ken worked in many ways to support this new department, including raising funds to build the Life Sciences building to house it. Another of Ken’s efforts was in support of the Molecular Biosciences Graduate Program, formed from a number of graduate programs in the life sciences at Rutgers and UMDNJ (New Brunswick). The programs consolidated their first-year fellowships and acquired additional ones from the Rutgers Graduate School; these were then used to recruit students into a first-class first year program involving an advanced curriculum and laboratory rotations. This program still exists today and is a model for graduate programs at universities across the country.

## 16. Patricia Morton, Associate Professor

Dr. Kenneth Breslauer: leader, colleague, mentor, and most of all, treasured friend. He always has time, always has good advice, and always has your back. Ken is a visionary: he sees beyond what others see, then builds a path which he enables everyone to traverse. He is a unique balance between dreamer and clear-eyed practitioner.

I came to Rutgers from outside academia, and thus into a world of different processes and practices. Dr. Breslauer took me under his wing and, more than once, guided me onto a safe and effective route, thus avoiding many invisible landmines.

He immediately understood the vision and commitment of our new W. M. Keck Center and was amazingly supportive as we tried new ways to raise funds, such as the very successful ‘CURE’ events in New York City; initiated programs across the normal lines of operations like the Presidential Lecture Series; and absolutely radically welcomed people with spinal cord injuries into our Research Center. His appearance at events was always very meaningful to people in the community, as they were honored that someone in his position would take time to speak to and be with them.

There are many stories I could tell but will sum things up by saying: The world would be a better, more creative, and genuinely caring place if there were more people like Ken Breslauer.

I am deeply grateful that Dr. Kenneth Breslauer has been, and is, part of my life.

## 17. G. Eric Plum, Lecturer

For more than 30 years, I have benefitted from Ken Breslauer’s mentorship and friendship. We all celebrate his stellar career as a scientist and university administrator. In addition to his intellect, Ken exhibits a rare gift for personal engagement and empathy. Everyone who worked with him appreciated his sensitivity about curveballs—although it was my understanding that he could not hit one.

The characteristic of Ken’s that I still find most remarkable is his enthusiasm about science. Despite his many conflicting responsibilities, he was always eager to discuss my latest ideas and experimental results. Early in my time in his laboratory, Ken and I made a trip to Long Island to confer with our collaborators at SUNY Stonybrook. Unlike the graduate students, who all had pretty nice cars, Ken drove an old Volvo with at least one wheel in the junkyard. The weather was bad and the Volvo’s wipers barely cleared the windshield of rain. I was quite unnerved. Throughout the trip, Ken exuberantly discussed the project with me while occasionally looking at the road. At that point, I believed that “I could do some really interesting science with this guy”—so long as we make it back to New Jersey.

Congratulations Ken on reaching this milestone, and thanks for the “interesting science” and your friendship over the years.

## 18. Jamshid Rabii, Professor Emeritus

My 33 years at Rutgers University have left me with many fond memories. Like many of my colleagues, a number of these memories stem from my experiences in research, teaching, as well as mentoring undergraduate and graduate students. In my case, however, I was fortunate enough to be encouraged by Ken Breslauer to join his team when he undertook development of the newly created Division of Life Sciences. That Ken was extremely successful in creating a world-class division engaged in research and teaching is a well-established fact. What may be less obvious is his invaluable influence on the professional lives of his team members. I recall accepting Ken’s offer of the position of Director of Undergraduate Affairs in the Division of Life Sciences with a degree of apprehension. Although at that time I knew Ken casually from being on several committees with him, I was not familiar with either his leadership qualities or his work ethics. It did not take very long for me to appreciate his impressive leadership ability and management style. It quickly became evident to me that Ken took genuine interest in all aspects of the Division’s development and performance and, when needed, offered his support and advice to every member of his team. Adding an ever-present composure and a good sense of humor to his other qualities made Ken Breslauer the ideal “boss.” There were many challenges along the way as I strived to manage the undergraduate affairs of the Division up to the high standards that were expected by Ken. It would have been near impossible to get through such challenges had it not been for Ken’s guidance and encouragement. Ken Breslauer’s support and mentorship, as well as his friendship, was an integral part of my career advancement at Rutgers University. I would be remiss if I did not mention that Ken’s support of his team members went beyond their professional activities within the Divison of Life Sciences. On more than one occasion, when frustrated in dealing with the local medical community for a health-related issue for myself or my family, all I needed was to reach out to Ken and he invariably employed his vast range of contacts to remedy the situation.

I am proud to have had Ken Breslauer as a boss, a mentor, and, especially, as a good friend.

## 19. Glen Ramsay, CEO

When Jack Aviv first introduced to me to Ken Breslauer, I was at that awkward stage of my professional life: transitioning from a postdoctoral fellow to my career. Career choices are a matter of self-preparation but also of opportunities created by others. I was, and still am, a bit of a duck: not the best flyer, nor swimmer, nor walker, but capable of all and of spanning the interfaces. Not being the best can stymie a career, but Ken and Jack saw an opportunity in me. Ken’s lab needed not only the best people, but also top-notch instrumentation. Jack used his companies to keep Ken well stocked with the necessary hardware. It was into this mutually beneficial relationship that I landed.

The job offer made to me, which I readily accepted, was a joint position in both of their institutions. The position was uniquely created for me, which I’m sure was an administrative feat. My work involved developing instrumentation that could benefit both men’s aspirations. The point I wish to make is that it was these men’s faith in myself (and others) that is supreme among their attributes. They always recognized that the collective advancement of others would ultimately be beneficial to all. This “faith in others”, I believe, is a chief contributor to their own successes. The result has been the launching of numerous careers, a huge number of publications, and, ultimately, improvement in our society.

This journal’s many articles and kind words are proof of Ken’s scope of influence. But let my experience demonstrate the depth of his generosity and commitment.

## 20. Armen Sarvazyan, CSO

Throughout his career, Ken Breslauer has been a leading figure in the field of thermodynamics of nucleic acids and their complexes. As a true visionary, Ken was quick to recognize the benefits of combining traditional calorimetric investigations with volumetric studies based on ultrasonic velocimetric and densimetric techniques. This combination was the foundation of an extremely fruitful collaboration between Ken, my former graduate student Tigran Chalikian, and myself. The collaboration is still ongoing, resulting in dozens of papers that provide unique insights into the physical nature of inter- and intramolecular interactions governing the biological function of proteins and nucleic acids. I want to use this occasion to wish Ken good health and continued scientific leadership for many years to come.

## 21. Jay A. Tischfield, Ph.D., FFACMG, Professor and Founding CEO

Ken Breslauer is an internationally renowned scientist and academic administrator, but perhaps most importantly, he is a visionary and builder of colleagues and programs. Ken and I had an extraordinarily productive partnership at Rutgers over a 20-year period, during which time I learned of and often appropriated the unorthodox ways in which he conducted his decadal role in the Division of Life Sciences and later as University Vice President for Health Science Partnerships. He taught me to act first and apologize later when we had a good idea that others ignored, frequently suborning co-conspirators whose interests coincided with ours. In retrospect, some of these individuals had divergent goals but they cooperated based on our pledge to support their interests in the future. We could do this because Ken’s word and handshake were viewed as his bond, no paper necessary in most instances, a characteristic that I seek to emulate.

Ken was the key individual responsible for recruiting me to Rutgers as the first permanent chair of the Department of Genetics. Initially, I did not want to take the job because I had spent the previous 25 years in various positions at schools of medicine and undergraduate programs were too intimidating. But then Ken did the most unusual thing: he telephoned my childhood friends who were still living in the New York metropolitan area, imploring them to convince me to come back to the region where I spent the first 21 years of my life. I was so impressed that anyone would have the imagination and chutzpah to do this, which clearly required a good spiel and the force of a persevering personality, that I came to Rutgers in 1998. I thought to build faculty research laboratories in Nelson Labs, but it proved to be wholly inadequate for the plans that Ken and I incubated. With unerring technique, Ken convinced the University administrative hierarchy to lend me the money to renovate space for what became the Rutgers Cell and DNA Repository (RUCDR). The project took off, and I was able to quickly repay the money. At every opportunity, Ken touted my success, knowing full well that we could use it as a launch pad for our greater goals. Next, he convinced the administration to build the Life Sciences Building at a time when there was very little construction on campus. In doing this, Ken demonstrated deftness and agility at maneuvering within the Rutgers political system. Almost everybody in the administration succumbed to the promises emanating from his velvet tongue. This was followed by a series of highly original agreements that Ken brokered between the University and RUCDR, creating the wealth and infrastructure (e.g., more buildings and equipment) that allowed RUCDR to deliver hundreds of millions of dollars of federal and industry grants and contracts, providing employment for well over a hundred people and recent privatization at a significant profit to Rutgers.

I stand in awe of Ken’s fundamental scientific contributions. His publications describe advances in our understanding of the thermodynamics of nucleic acids, a subject that I can only approach at 30,000 feet. My understanding of some of the biophysical principles that Ken describes in his papers is a tribute to their fundamental importance and his clear and highly articulate writing. I am honored that he has occasionally solicited my input or opinion, especially as it relates to medical implications.

Ken and I were both raised in New York City, though we occasionally regard our home boroughs as different planets. Oddly, we were both at Yale for graduate studies at the same time, though we didn’t know each other because he was in the Department of Biophysics with the smart people. In fact, I almost flunked the graduate-level physical chemistry course offered by Ken’s advisor. After Yale, we both gravitated to the University of California at Berkeley for postdoctoral studies, but we never met until he led the Rutgers recruitment dance.

I pay homage to Ken Breslauer on his 75th birthday, though I’ve been a fan and acolyte since we first met. I thank him for being a great friend and remarkable human being and for teaching me to navigate the Rutgers system. I regret that our paths did not cross before I joined Rutgers, especially in view of our prior geographic proximity.

## 22. Douglas H. Turner, Professor Emeritus

Ken and I were postdocs together in Nacho Tinoco’s lab for about 20 months in 1973–1974. We were both Yankee fans from New York, so we bonded immediately. He has been a good friend ever since. At Berkeley, he reminded me of Mickey Mantle because he was the best softball center fielder I ever saw, especially his breaks on fly balls. We also bonded on watching Monday Night Football games. Along with Eric Weitz (now at Northwestern University), we were the greatest chefs of TV dinners on those nights. After Ken was set up at Rutgers, he kindly let me, and Sue Freier (now at IONIS Pharmaceuticals), come to his lab for a series of calorimetry experiments on poly-C. Together with Luis Marky, the four of us pioneered high throughput calorimetry by perfecting sleeping on the lab floor while experiments were running. Ken’s calorimetry has had a major effect on my career due to his 1975 JMB publication with Sturtevant and Tinoco establishing that optical melting experiments best agree with calorimetry when linear lower baselines are subtracted from the UV absorption curves. My group has applied this crucial insight in at least 60 papers.

From later visits, it became clear that Rutgers was lucky that Ken expanded his interests to include academic administration. Our original bonding with the Yankees was also important for those visits. When Ken gave me directions for driving to his house, he said, “When you come to a fork in the road, take it.” Turns out Ken lived near Yogi Berra, and Yogi famously gave the same directions, but expanded them to include living life. That made them easy for me to remember.

## 23. Jens Völker, Associate Research Professor

It is a great honor and privilege, and no easy task, to be involved in editing a Festschrift/Special Issue on the occasion of Professor Kenneth J. Breslauer’s milestone birthday and to come up, in a few words, with something to say about Ken. Where does one start with someone as multifaceted as Ken Breslauer, and how does one keep it to just a few words?

Perhaps it is best to start where it all began for me, as my first introduction to Ken speaks volumes about the kind of person Ken is, beyond his numerous scientific and administrative achievements. I first met Ken when I attended, as a lowly and very much overawed PhD student from a far corner of the earth, the Gordon Conference on Biopolymers Ken co-chaired in 1992. I met him by literally bumping into him and a group of invited speakers and other luminaries in the elevator of the conference center. Ken immediately turned around and introduced himself by saying, “I thought I knew most people at the conference. I don’t know you. I am Ken Breslauer.” Upon hearing I was the odd PhD student from South Africa (we had communicated by fax so that I could get an invite to the conference), Ken proceeded to ask what my PhD was all about (thermodynamics of DNA triple helices), and if I had a poster at the conference. Upon hearing that indeed I had, he turned to his colleagues and said, “Carry on without me, I need to see this” and proceeded to spend the next hour and then some talking with me about my PhD project and invited me to visit his lab, if I got the chance. In the process of talking to me, whether intentionally or not I do not know, he managed to greatly alleviate my considerable anxieties of how my research done in isolation at the far end of the world compared with world-class research done in the US. It is this ability to talk to people from all walks of life and to make them feel comfortable I have since come to value and appreciate as one of Ken’s greatest assets, a view that is also reflected in the numerous personal reminiscences listed in this editorial and the wide range of authors willing to contribute to this Special Issue.

Two years later, I accepted a postdoc position in Ken’s lab, and I have been collaborating with Ken ever since. Having worked with Ken (often in collaboration with my former PhD advisor Horst Klump until his untimely passing last year), I have since come to appreciate many of Ken’s other qualities, such as his willingness and encouragement to let me pursue my data to wherever they may lead (however outlandish it initially might have seemed); his patience with my struggles to make sense of my data and write them up in a semi-coherent manner (and whose importance he seemed to grasp within minutes when I finally felt comfortable to share them); his ability to convert my semi-coherent texts into something even a non-specialist can follow; and, not in the least, his willingness to find time to deal with research results despite his full administrative plate as dean and vice president at Rutgers. (Even if it sometimes took a while for him to get around to read/edit the many manuscripts produced in the lab—a standing joke in the lab for many years was that KJB (Ken’s Journal of Biology) was a most exclusive journal with a readership of ONE, containing the hottest results unavailable to anyone else.) However, despite all these (and many other) academic/professional achievements, it is my opinion that Ken’s ability to relate to people, to take their concerns seriously, and, when needed, his unstinting willingness to help out when life throws a curveball (as several other contributors have commented and that I can attest to from personal experience) that represent Ken’s greatest gifts and that make it such an honor and privilege to have been involved in this Special Issue and to write this testimonial.

Happy Birthday Ken, and I hope we will find many an interesting and fruitful research topic to pursue in the years to come.

## 24. Peter von Hippel, Professor

I send my enthusiastic best wishes on the occasion of this celebration of your 75th birthday by your many friends, colleagues, and admirers. Achieving this three-quarter-century mark represents a momentous milestone in your busy and productive personal and scientific life and provides a point at which one could legitimately consider ‘hanging up one’s boots’. However, you show no signs of slowing down, and therefore, this celebration and Festschrift could be considered instead as a ‘mid-career assessment’ opportunity for you, an opportunity for your friends and colleagues to celebrate all that you have accomplished, and an opportunity to help you think about what you might still want to attempt in the ‘second-half’ of your scientific career.

I had hoped to mark the occasion by providing an overview of some recent and (thanks to the new methods developed by my research collaborators) perhaps ‘ground-breaking’ single molecule and 2D fluorescence spectroscopy work we have been conducting on the structural characterization and rates of inter-conversion of single-stranded (ss)DNA conformations on microsecond time scales, which has also helped us to more directly approach issues involving how various DNA conformations actually interact with ssDNA-binding proteins in real time. Clearly, this work builds on the major calorimetric and other biophysical chemical studies of DNA (and RNA) structures and sequences and their interactions with binding ligands that you and your lab have been engaged in for so many years. In our recent studies, we have been able to use some new methodologies to actually begin to monitor the appearance and disappearance of elements of the secondary structure of ssDNA on microsecond time scales, rather than just inferring (through monitoring the slower protein rearrangements that follow) what the DNA must be doing at rates that have been too fast to observe directly. We have also been studying the fluctuations in and the balance of forces that control the structure, stability, and inter-conversion rates of double-stranded DNA, and the fork and ss–dsDNA junctions that connect ds- and ssDNA segments in the DNA scaffolds of replication and transcription complexes. These latter loci serve as the positions at which the protein components of these systems actually function. This is, of course, a field to which you and your collaborators have contributed much, and thus such a paper seemed particularly appropriate for your Festschrift. However, now that the deadline is actually upon us, I find that I have, unfortunately (and not for the first time), over-estimated the rate at which this research could proceed before a review could legitimately be written. Therefore, our contribution will have to be limited to this shorter congratulatory message.

I close with a few more personal thoughts. We have been friends and have interacted, both scientifically and personally, for many years. In that capacity, I have much enjoyed reading and thinking about (and on occasion, serving as editor of or reviewer for) the seminal scientific work that you and your collaborators have produced. Those studies have mostly used physical biochemical techniques to characterize the structures and interactions of nucleic acid components with one another and with the proteins that drive the various processes of genome expression at the cellular and evolutionary levels. Your group’s work has resulted in the production of unique and invaluable databases that have helped to put nucleic acid studies on a sound thermodynamic footing and have provided the necessary parameters to facilitate the discovery of ligands and drugs that have both increased our basic knowledge and have helped to devise rational approaches to the treatment of various diseases. You have also served as an insightful and constructive editor and reviewer for our papers. I can’t now remember exactly when we first met, but our paths have crossed in multiple contexts, including formal and informal occasions connected with basic and applied research issues at meetings and seminars and visits and correspondence over at least the last 40 years. I look forward to the next 40! My colleagues and I are grateful for your many scientific and administrative and personal contributions to our field and to our lives. Thank you!

## 25. Gabrielle Wilders, Senior Executive Associate

I have worked with Dr. Breslauer for several years at the School of Arts and Sciences. He frequently goes out of his way get to know the staff, tell a joke, or share a story.

When I was diagnosed with a serious medical illness, I was in a state of shock and unsure of next steps. I reached out to Dr. Breslauer for help. I knew he appreciated the urgency of my situation; I knew he would know the top doctors in the area, and most importantly, I knew that helping his friends and colleagues in these situations was a priority to him.

Without hesitation, he paved the way for me to see leading experts in the field until I found one that I was completely comfortable with. I would not have been able to navigate my way to the excellent care I received without Dr. Breslauer’s skilled guidance and compassion.

I am eternally grateful for the kindness he has shown me and the concerted effort he put forth to help ensure that I regained my health.
